# LIFE PROBLEMS, DAILY ACTIVITIES, AND ALERTNESS IN LATE LIFE

**DOI:** 10.1093/geroni/igae098.2371

**Published:** 2024-12-31

**Authors:** Sibo Gao, Zexi Zhou, Karen Fingerman

**Affiliations:** The University of Texas Austin, Austin, Texas, United States; The University of Texas Austin, Austin, Texas, United States; The University of Texas Austin, Austin, Texas, United States

## Abstract

Older adults facing life problems often report worse psychological and physical well-being compared to their counterparts without such issues. Daily activities may play an important role in these links. This study examined the association between life problems, daily activities, and alertness (i.e., being energetic and feeling less tired) in late life. We used the data from the Daily Experiences and Well-being Study. Participants aged 65+ (Mage=74.15, N=258) indicated whether they had experienced life problems (e.g., health, financial, legal) in the past year. Every 3 hours for 5–6 days, participants completed ecological momentary assessments where they reported engagement in various daily activities (e.g., eating, driving, doing chores) and level of alertness. They also wore Actical accelerometers to record their physical activities across the study period. Multilevel models revealed that participants with more life problems reported a similar amount of physical activities (i.e., sedentary time, energy expenditure), but lower levels of alertness (i.e., feeling less energetic and more tired) throughout the day, compared to those with fewer problems. Participants with more life problems reported feeling more energetic in assessments when they engaged in more leisure activities (e.g., reading, watching TV) compared to assessments when they engaged in less. In contrast, for participants with fewer life problems, leisure activities were not associated with the feeling of being energetic. Findings suggest that older adults with more life problems may be more vulnerable in everyday alertness, and emphasize the need to consider the salubrious effect of leisure activities amid problems in late life.

